# Assisted dying: principles, possibilities, and practicalities. An English physician’s perspective

**DOI:** 10.1186/s12904-024-01422-6

**Published:** 2024-04-13

**Authors:** Robert Twycross

**Affiliations:** 1 Emeritus Clinical Reader in Palliative Medicine, Oxford University, Oxford, UK; 2 Sir Michael Sobell House, Churchill Hospital, Old Rd, Headington, Oxford, OX3 7LE UK

**Keywords:** Assisted dying, Physician assisted suicide, Autonomy, Disinformation, England and wales, Vulnerability

## Abstract

It seems probable that some form of medically-assisted dying will become legal in England and Wales in the foreseeable future. Assisted dying Bills are at various stages of preparation in surrounding jurisdictions (Scotland, Republic of Ireland, Isle of Man, Jersey), and activists campaign unceasingly for a change in the law in England and Wales. There is generally uncritical supportive media coverage, and individual autonomy is seen as the unassailable trump card: ‘my life, my death’.

However, devising a law which is ‘fit for purpose’ is not an easy matter. The challenge is to achieve an appropriate balance between compassion and patient autonomy on the one hand, and respect for human life generally and medical autonomy on the other. More people should benefit from a change in the law than be harmed. In relation to medically-assisted dying, this may not be possible. Protecting the vulnerable is a key issue. Likewise, not impacting negatively on societal attitudes towards the disabled and frail elderly, particularly those with dementia.

This paper compares three existing models of physician-assisted suicide: Switzerland, Oregon (USA), and Victoria (Australia). Vulnerability and autonomy are discussed, and concern expressed about the biased nature of much of the advocacy for assisted dying, tantamount to disinformation. A ‘hidden’ danger of assisted dying is noted, namely, increased suffering as more patients decline referral to palliative-hospice care because they fear they will be ‘drugged to death’.

Finally, suggestions are made for a possible ‘least worse’ way forward. One solution would seem to be for physician-assisted suicide to be the responsibility of a stand-alone *Department for Assisted Dying* overseen by lawyers or judges and operated by technicians. Doctors would be required only to confirm a patient’s medical eligibility. Palliative-hospice care should definitely *not* be involved, and healthcare professionals must have an inviolable right to opt out of involvement. There is also an urgent need to improve the provision of care for all terminally ill patients.

## Background

The Parliamentary Office of Science and Technology in the United Kingdom (UK) defines Assisted Dying (AD) as:

The involvement of healthcare professionals in the provision of lethal drugs intended to end a patient’s life at their voluntary request, subject to eligibility criteria and safeguards. It includes healthcare professionals prescribing lethal drugs for the patient to self-administer (‘physician-assisted suicide’) and healthcare professionals administering lethal drugs (‘euthanasia’) [1].

This reflects the definitions used in medical and ethical literature, and will be used in this paper. However, confusingly, the pro-AD organization *Dignity in Dying* (https://www.dignityindying.org.uk/) limits AD to physician-assisted suicide (PAS) in patients with a prognosis of less than 6 months. Equally confusing is the decision by the House of Commons Health and Social Care Committee (UK) to use of the term ‘assisted dying/assisted suicide’ (AD/AS) when talking about any type of physician-assisted death [2].

Historically, the demand for AD stems from the fact that the suffering of terminally ill people is not always relieved and, for some people, there is a level of existence below which they would wish to die. At present, AD is available in all or parts of around 12 countries [3], amounting to about 4% of the world’s population. In some, both PAS and euthanasia are permitted, in others just PAS. Eligibility criteria and safeguards vary.

This article focuses on England and Wales (E&W) where, over the last 20 years, numerous attempts have been made to legalize PAS. For those with reservations about such developments, it may seem that they have only two choices: either to ‘go with the flow’ or, conversely, actively campaign against any form of AD. However, there is a third option: active involvement in the debate, seeking positively to influence any proposed legislation. Devising a law which is ‘fit for purpose’ is definitely *not* an easy matter [4]. Existing AD laws are not uniform, and the consequences of legislation will depend on the model under consideration [3].

The over-riding utilitarian consideration is that more people should benefit from a change in the law than be harmed. *In relation to AD, this may not be possible.* At the very least, any AD law must aim to achieve an appropriate balance between compassion and patient autonomy on the one hand, and respect for human life generally and medical autonomy on the other. Protecting the vulnerable is a key issue. Likewise, not impacting negatively on societal attitudes towards the disabled and frail elderly, particularly those with dementia.

### The present situation

Although the inquiry by the Health and Social Care Committee of the House of Commons in 2023 extended to AD generally [2], for more than 20 years all the Bills introduced into Parliament have been limited to PAS. For a cluster of reasons, it seems that some form of PAS is likely to become legal in E&W within the next few years. The very supportive media coverage gives the impression that PAS is ‘a concept whose time has come’. Pro-AD activists claim that there is overwhelming public support. Every few months, a celebrity announces their intention to avail themselves of the services of *Dignitas* in Switzerland when ‘the time comes’, with renewed extensive media attention. Individual autonomy (‘self-rule’) is regarded as an unassailable trump card: ‘my life, my death’. AD Bills are at various stages of preparation in neighbouring jurisdictions (Scotland, Republic of Ireland, Isle of Man, Jersey); this adds to the growing sense of inevitability [5–8].

Some surveys have suggested that over 80% of the population in the UK are in favour of AD, although a recent one limited to PAS gave the lower figure of 65% [9]. It should also be noted that, in a survey in 2021 on behalf of the UK All-Party Parliamentary Group for Dying Well, 10% of respondents thought AD meant providing hospice-type care to people who are dying, and 42% that it meant giving people who are dying the right to stop life-prolonging treatment. *Fewer than half (43%) of respondents knew what the term ‘assisted dying’ actually meant* [10]. This suggests that claims about the level of public support for AD should be interpreted with caution.

### Existing models of physician-assisted suicide (PAS)

Switzerland, Oregon (USA), and Victoria (Australia) represent three models of PAS. In the USA, although the laws may not be completely identical in other states where PAS is permitted, they are all based on Oregon’s. Likewise in Australia, they are based on Victoria’s. Benelux and Canada will *not* be discussed because, in those countries, AD is almost always euthanasia.

In all three models, a doctor prescribes the lethal prescription after confirming the person has mental capacity, is aware of alternatives such as palliative-hospice care (PHC), the request is enduring, was not made under duress, and that the medical eligibility criteria are met. But in other respects, the models differ. In Switzerland, there is no prognostic limit, and no residency requirement. Suffering is not specifically mentioned in Oregon, just a prognosis of less than six months. Following a federal lawsuit in 2022, residency is no longer a requirement. In Victoria, residency and both suffering and a limited prognosis (generally six months but 12 months for neurodegenerative conditions) are prerequisites. The number of safeguards increases progressively across the three models.

#### Switzerland

In Switzerland, although four out of 26 cantons have laws concerning access to PAS in healthcare institutions, there is no federal law. However, it is possible throughout the country because of the wording in the Swiss Criminal Code (1942) which states that an offence is committed only if assistance is *for selfish motives*. In the absence of such motives, the assisting person is *not* criminally liable. Taking advantage of this loophole, two not-for-profit organisations called *EXIT* (German- and French-speaking, respectively) were set up in 1982 to facilitate PAS for residents in Switzerland with incurable progressive disease. Subsequently, *Dignitas* was set up to meet the needs of non-residents.

The Swiss Academy of Medical Sciences (SAMS) provides ethical guidance to doctors in its document *Management of Dying and Death* (revised 2022) [11]. The section on PAS is helpfully discussed within the general context of care of the dying. For example, it is stressed that:

‘The true role of physicians in the management of dying and death… involves relieving symptoms and supporting the patient. Their responsibilities do not include offering assisted suicide, nor are they obliged to perform it. Assisted suicide is not a medical action to which patients could claim to be entitled, even if it is a legally permissible activity’ [11].

The 2022 revised guidance has extended the eligibility for assisted suicide considerably. The former requirement that ‘the patient’s illness justifies the assumption that the end of life is near or can be expected to be near’ has been replaced by ‘the symptoms of disease and/or functional impairments are a source of intolerable suffering for the patient’ [11]. One reason for this change may be the fact that a review of practice from 1999 to 2018 indicated that over 50% of cases probably had not met the key criterion of a short life expectancy [12]. In this same period, the number of deaths by PAS rose steadily from 0.2 to 1.8% of all deaths [13].

For doctors willing to be involved, their role is limited to assessing the person’s decision-making capacity, confirming the constancy of their request, providing a statement about their medical condition, and subsequently prescribing a lethal dose of pentobarbital. The prescription is collected from the pharmacy by a volunteer from *EXIT* or *Dignitas* on the day of the assisted suicide. Most deaths take place at home or, in the case of *Dignitas*, in a room provided by the organisation. At present, because many hospices and palliative care units do not allow PAS on their premises, most patients return home for this. However, in French-speaking areas, hospitals increasingly allow PAS if a discharge is impractical. For those who return home, in case of a change of mind, their bed is kept available until confirmation of death has been received. Everything is carefully documented, and the police are notified immediately after the person has died.

#### Oregon

Oregon’s Death with Dignity Act (DWDA) came into effect in 1997 and is held up by some as an example of how a PAS law can be safely enacted – described as ‘tried and trusted’ by *Dignity in Dying*. However, an analysis of the annual reports issued by the Oregon Health Authority between 1998 and 2023 gives grounds for caution [14]. Indeed, the Danish Ethics Council concluded recently that the Oregon model is not ‘sufficiently clear in [its] delineations, fair in [its] justifications for access, or sound in terms of control mechanisms’ [3].

Originally the DWDA was limited to residents but, in 2022, a federal lawsuit (brought by an Oregon doctor) forced a change which allows non-residents to access PAS within the state [14]. The DWDA allows people ≥ 18 years of age diagnosed with a terminal illness and expected to die within six months to end their lives through the self-administration of a lethal dose of drugs prescribed by a doctor. A Coordinating doctor and a Consulting (specialist) doctor determine whether the person is medically eligible, is not acting under duress, and that the request is enduring. The Oregon Health Authority must be informed when a prescription is written by the Coordinating doctor. The lethal dose can be collected by the patient or their representative and kept at home until the patient decides that the time has come to take it – without further reference to their doctor. In this model, there is no reference to ‘intolerable suffering’.

Only about 2/3 of the issued prescriptions are used. In Oregon over 25 years, the three most frequently reported end-of-life concerns behind the request for PAS have been a decreasing ability to participate in enjoyable activities (90%), loss of autonomy (90%), and loss of dignity (72%) – all more existential than medical. Inadequate pain control, or concern about it, featured in only 28%. Most patients (92%) died at home, and 91% were enrolled in hospice care (mostly home-based in the USA), although the nature and extent of that care is not specified [15].

In 2022, 146 doctors wrote 431 lethal prescriptions (1–51 prescriptions per doctor; most just one or two). Prescribing doctors were present at the time of death for 13% of the patients; other healthcare providers for another 13%, and volunteers for 18%. One patient died in hospital, and one in a hospice facility. Where known, time from ingestion until death ranged from 3 min to nearly 3 days, with a median time of 52 min. Almost all involved the drug combination DDMA (diazepam, digoxin, morphine, amitriptyline) ± phenobarbital. In 2022, AS accounted for 0.6% of all deaths.

In 2017, an unsuccessful Bill was introduced to allow surrogates to administer the drugs to those who had subsequently lost decisional capacity after receiving a lethal prescription, with proposals to extend the DWDA to allow euthanasia for those with dementia and those incapable of swallowing drugs.

Concern has been expressed that most of those dying by PAS are clients of *Compassion and Choices*, the AD advocacy organisation in Oregon, and discussion of alternatives may have been limited, particularly as the association between the patient and the prescribing doctor is sometimes < 1 week (median 3 months). A review of five patients whose details are in the public domain revealed inadequate exploration of their concerns and a bias in favour of PAS [16]. Further, despite a known incidence of depression of up to 40% in those with a genuine desire to hasten death, only three patients (0.7%) in 2022 were referred for psychological or psychiatric evaluation. Some patients have delayed ingesting the lethal medication for 2–4 years, thereby emphasizing the difficulty of determining the likely prognosis.

#### Victoria, Australia

The Voluntary Assisted Dying (VAD) law came into effect in 2019 [17]. Unlike Oregon, legislators had the benefit of 20 years of experience in other countries where AD Bills have been introduced and/or laws passed. As in Oregon, two doctors are involved: the Coordinating doctor, who initially informs the person about end-of-life-care options and supports, then assesses the person’s eligibility, and whether the request is voluntary and enduring; and the Consulting doctor, who re-assesses the patient’s request. Both doctors must be either a vocational general practitioner or a member of a specialist college, and one must have at least five years post-fellowship experience and experience in the patient’s condition.

Institutions can forbid VAD on their premises, and involvement by doctors is voluntary. Those volunteering must undertake the mandatory online ‘approved assessment training’ required by the law before they can participate [18]. Of those across the state who have volunteered, around 300 have ‘currently active’ profiles, representing about 1% of the total medical workforce; 60% are General Practitioners (GPs) [17]. Much of the work is unremunerated. Coordinating an application through to a patient’s death can take up to 60 h of a doctor’s time. As a result, because of inevitable time constraints, some doctors report undertaking less than ideal assessments and/or not being able to see their other patients because of the VAD workload [19].

Although the primary focus is PAS, the law extends to euthanasia (‘practitioner administration’) if a person is incapable of swallowing the medication. In this case, the lethal drugs are administered intravenously by the Coordinating doctor, who can delegate this duty to the Consulting doctor subject to agreement by both parties. The person must have an advanced, progressive, incurable disease or medical condition that is expected to cause death within six months, or 12 months for neurodegenerative conditions, and is causing suffering that cannot be relieved to an extent considered tolerable to the person. The Victorian Government claims that, with 68 safeguards, the law is the safest and most conservative in the world [20]. However, the safeguards are not all aimed at patient safety; some, such as conscientious objection provisions, are explicitly labelled as ‘practitioner protections’. Although the process is complex, non-medical ‘Care Navigators’ are available to guide patients through the process. After initiating the process themselves, a person has to make three separate requests to end their life: an initial verbal request, a second written and witnessed request, and a final verbal request. The time between the first and final request must be at least 9 days.

The most striking safeguard is that doctors and other healthcare professionals are forbidden to initiate discussions about AD. They can only respond to a patient’s direct request for information. There is guidance as to what may or may not constitute a direct request, which needs to be specific and explicit. This is intended to avoid coercion or suggestion, but not to discourage discussion. Requests can only be initiated by the person and cannot be done via telehealth [18]. The *Voluntary Assisted Dying Statewide Pharmacy Service* have sole responsibility for checking for necessary authorisation permits, and preparing and supplying the lethal drugs. The cocktail of drugs is delivered in a locked box to eligible patients in their homes across the state, and a contact person is appointed who will be responsible for returning the medication if unused after the person has died. A list of instructions on how to mix and drink the lethal drugs are included. As in Oregon, people drink the lethal dose at a time of their choosing. In 2021–2022, AD deaths amounted to 0.58% of all deaths.

### Vulnerability

In October 2023, the Danish Ethics Council published a report in response to a request from the Danish Parliament’s Health Committee to issue a statement which could be included as part of the basis for the Danish Parliament’s discussions of and decision on the citizens’ proposal that there should be legislation permitting AD [3]. The Council concluded that: *‘The only thing that will be able to protect the lives and respect of those who are most vulnerable in society will be an unexceptional ban’*. It pointed out that AD risks causing unacceptable changes to basic norms in society, the health care system and human outlook more generally. The very existence of an offer of AD will decisively change ideas about old age, infirmity, dying, and quality of life. In the Council’s opinion, if AD becomes an option, there is too great a risk that it will become an expectation aimed at certain groups in society.

Vulnerability is seen in all strata of society, often stemming from social isolation and a sense of helplessness and hopelessness. The most vulnerable include those who feel a burden. Those in despair will be more likely to make a request for AD, and no law can prevent this. Vulnerability is also associated with a lack of continuity in care. When life is hard, everyone without exception needs *reliable, trustworthy human support* to enable them to cope. The most fundamental human fear is that we will be abandoned, and the corresponding fundamental hope is that we will not be. Non-abandonment is dependent on continuity of care – not just reactive but pro-active with, for example, a ‘hot-line’ to a named GP and/or nurse. Without this, patients feel abandoned and worthless; symptoms escalate coupled with a sense of despair. In contrast, continuity of care affirms to patients that they still matter and that they are still persons of worth.

Unfortunately, continuity of care is increasingly difficult to access in the UK now that the National Health Service (NHS) is understaffed and overworked, particularly in Primary Care. In recent years, access to one’s named GP has become much harder, sometimes impossible. Arranging an appointment has generally become more complicated – now often necessitating an online request, to be ‘triaged’, with a response promised within 24 h. It is challenging and off-putting. Without ready access, people feel abandoned and hopeless, resulting in despair.

Some of the most vocal opponents of PAS are among the disabled and disability associations because they fear that its availability, even if limited to the terminally ill, will have a negative impact on attitudes to disability. ‘Disableism’ is rife in Britain, with its tendency to value the worth of a life in terms of its economic utility to society. PAS could well lead to a further narrowing down of what is viewed as a liveable and dignified life, with some people’s lives being considered worthless, merely an economic drain on society’s resources. Even in the UK, access to quality PHC is still patchy. Pressure to opt for AD would most likely increase as the health budget becomes further squeezed. Based on experience in other countries, it is naïve to believe that an incremental widening of the eligibility criteria will not happen. In this connection, it should be noted that, in the Netherlands, the mainstream political parties have expressed support for the *Completed Life Initiative* [21]. If this became law, this would permit euthanasia for those over the age of 74 who are ‘tired of life’. Thus, legalising AD should not simply be regarded as a small step to bring relief to a few. In terms of possible unwanted consequences, it is a massive step.

A further problem is the inability of many doctors to relinquish the goal of ‘fixing the problem’. This can lead to a feeling of failure, and an inclination to withdraw – with death seen as the only way to deal with the suffering. Doctors who cannot switch from a cure to a comfort *modus operandi* when it is appropriate may well unconsciously coerce patients towards AD. There are numerous anecdotes supporting this contention [22]. Such unwitting abuse is also linked to unconscious bias stemming from the doctor’s own fear of death [23]. It will become a bigger problem if the expectation shifts towards routinely informing potentially eligible patients about AD as one of their options to be considered. In Canada, the *Canadian Association of MAiD Assessors and Providers* (CAMAP) recommends that all who might qualify for MAiD (Medical Assistance in Dying) should be told about it as an option [24]. Offering MAiD to a patient who has not raised it could be interpreted as meaning that their suffering is likely to become intolerable, and that MAiD is the recommended way out, thereby impacting negatively on the patient’s resilience. On the other hand, a total prohibition on raising the subject, as in Victoria, could prevent someone from exercising their legal right through ignorance [25].

### Hidden danger: AD resulting in more suffering?

Even in the absence of AD, some people decline referral to PHC despite unrelieved pain and/or other distressing symptoms because they fear they will be ‘drugged to death’. This is a real phenomenon well-known to PHC professionals (in fact, PHC typically prolongs survival by weeks or months as a result of, *inter alia*, improved comfort, sleep, and appetite.) This unfounded fear will most likely be enhanced if AD is legalized, particularly if PHC is involved.

### Autonomy

Autonomy in medicine is based on *a partnership between doctors and patients, each respecting the autonomy of the other* [26]. This relational model of decision-making incorporates mutual respect and trust, dialogue, and informed negotiation. It contrasts with ‘consumer autonomy’ where the patient demands specific interventions from the doctor, regardless of established medical norms. In this scenario, the doctor is reduced to being an agent for carrying out a patient’s preferences (‘my legal right’) – a technician, no longer a professional. It becomes a transactional relationship (that of purchaser and supplier) rather than a partnership.

It is generally accepted that people have the right to self-determination provided their actions do not harm others. However, exercising personal autonomy (‘self-rule’) means making a choice. Informed choice requires reliable information about relevant options. Without this, autonomy is not valid. Disturbingly, official reports about AD do not specify the nature of the PHC received by those opting for AD. What is known is that to varying degrees there is poor access to palliative care in all the jurisdictions where AD is permitted. For example, in Canada, only half of the population are able access any form of palliative care [27].

Further, the extent to which an autonomously expressed wish for AD should be acted on must be balanced against the rights of the other people involved, notably the family and health professionals. From a medical point of view, AD will always be a ‘last resort’ option: a patient must be suffering from intractable symptoms and/or functional impairment caused by an incurable disease with no realistic expectation of relief within an acceptable time frame. Medical involvement would be unethical, for example, in healthy elderly people simply ‘tired of life’ [11].

It is imperative that conscientious objection by doctors and other healthcare professionals is guaranteed, as in the three models of AS described above. Lord Joffe, who introduced two AD Bills in the House of Lords 15–20 years ago, is reputed to have said that, if doctors objected to AD, they should be forced to comply, thereby revealing a total misunderstanding of the doctor’s professional role. *Such high-handedness feels threatening*. If Lord Joffe’s suggestion was adopted, it would turn doctors into technicians. In 2012, the White Paper, *Equity and Excellence: Liberating the NHS* set out a vision of a health service which puts patients and the public first, where ‘no decision about me, without me’ is the norm. It included proposals to give patients more say over their care and treatment with more opportunity to make informed autonomous choices. The slogan should be applied equally to doctors in relation to AD. Indeed, the British Medical Association (BMA) states that any legislation to permit AD should be based on an ‘opt-in’ model, so that only those doctors who positively choose to participate can do so. Doctors who opt in to provide the service should also be able to choose which parts of the service they are willing to provide (e.g. assessing eligibility and/or prescribing and/or administering drugs to eligible patients) [28].

In 2020, the BMA surveyed its members about their attitudes to AD [29]. The response rate was only 19%, thereby casting doubt on whether the results truly represent the views of the membership. In the answers to ‘In principle, do you support or oppose a change in the law to permit doctors to prescribe drugs for eligible patients to self-administer to end their own life?’, 50% said they would support, 39% would oppose, and 11% were undecided. (For administration by doctors (euthanasia), only 37% were in favour, 46% against, 17% undecided.) When broken down into specialties, in relation to AS, Palliative Medicine doctors were 76% against, 14% in favour, and 10% undecided. There were also majorities against in Clinical and Medical Oncology, Gastroenterology, Geriatric Medicine, Renal Medicine, Respiratory Medicine, and General Practice [29, pp. 103–105].

In contrast, respondents who had voted for legal change contained a majority of retired doctors, medical students and those in specialties which involve little or no contact with terminally or otherwise incurably ill patients. It seems that *the more doctors are involved in caring for dying patients, the greater the likelihood that they will oppose a change in the law.* Further, in relation to AS, when asked if they would personally be willing to participate, a majority said they would not (45% vs. 36%, and 19% undecided) [29]. Previously in 2019, in a survey by the Royal College of Physicians of London, 85% of Palliative medicine doctors were against a change in the law, a similar number said they would refuse to participate in AD, and only 5% were willing to assist suicide themselves [30].

These figures may seem bizarre. Why should doctors working in PHC be those most strongly against a change in the law, and would want not to be involved? After all, these are the doctors most likely to witness suffering at the end of life. It is often claimed that opposition to AD stems from blindly accepted religious dogma. However, as in other areas of medicine, many palliative care doctors are not religious. (And don’t forget *Humanists against Assisted Suicide and Euthanasia* [31]). But whatever the reasons, the fact remains that most PHC doctors are against a change in the law and would strongly object if any form of AD was integrated into PHC.

Perhaps the answer to this conundrum can be found in a recent Swiss study of PHC doctors, ‘*How is it possible that at times we can be physicians and at times assistants in suicide?*’ [32]. All the doctors interviewed stated that PHC and assisted suicide are diametrically opposed approaches, based on different philosophies. In PHC, there is a commitment to *non-abandonment*: ‘Whatever happens, we will stay beside you every step of the way. Together we will get through this’. *Compassionate presence* and *compassionate listening* together demonstrate that *the patient still matters and is still a person of worth.* This is the essence of palliative care. It lightens the patient’s load of cares by, *inter alia*, decreasing their isolation and sense of worthlessness. In such an environment almost all patients lose their wish to hasten death. This is the essence of PHC, and it is difficult to switch to an alternative approach. *It is difficult, if not impossible, to work looking in two diametrically opposite directions*.

## Disinformation

Regrettably, those campaigning for AD consistently underestimate the potential harms associated with a change in the law [4, 24]. Further, using the phrase ‘dying with dignity’ to describe AD, although a brilliant tactical move, is tantamount to disinformation. Likewise, naming the PAS statute in Oregon as the ‘Death with Dignity Act’. These phrases are also widely used in the media. As a result, many people now imagine that anything other than AD will be extremely distressing and undignified. In fact, ‘dying with dignity’ is equally applicable to quality end-of-life care. ‘Dignity in care’ is used as a way of describing the human side of medical care generally – with many scientific studies demonstrating what dignity means to patients and ways of enhancing it [33].

The choice of patients’ stories used to support the case for AD is also disturbing. Some are clear examples of poor care. If poor care is driving people to contemplate AD, this is not a truly autonomous decision, but one forced on the patient because of the absence of choice. Equally upsetting have been examples of patients with motor neurone disease/amyotrophic lateral sclerosis (MND/ALS). It has been suggested that the only alternative to AD is suffocating to death in great distress when non-invasive assisted ventilation (NIV) is discontinued at the patient’s request. This is fearmongering and causes great unnecessary distress. There is a well-established protocol for individually-tailored anticipatory sedation to allow a patient to be unaware while they die following the removal of assisted ventilation [34].

### Seeking the ‘least worse’ option

Given the widespread disquiet felt by doctors, *a law with minimal medical involvement would be the most equitable*, such as in Switzerland and Oregon. This makes good sense given that the three most frequently reported end-of-life concerns behind the request for PAS in Oregon are more existential than medical: a decreasing ability to participate in enjoyable activities (90%), loss of autonomy (90%), and loss of dignity (72%) [15]. The Swiss model is unlikely to appeal to legislators in E&W, even though theoretically it could simply mean changing the Suicide Act 1961. At present this states that, without exception, it is an offence to assist or encourage another person’s suicide.

The most recent PAS Bill introduced in the UK Parliament was in 2021 by Baroness Meacher in the House of Lords. A novel feature in the Bill was the need for the patient’s eligibility and voluntary request to be ratified by the High Court (Family Division) before the lethal drug(s) could be dispensed. As in Oregon, there was no mention of suffering, just a terminal illness with an expected prognosis of under six months. Psychiatric illness alone, including depression, was specifically excluded as an eligibility criterion. However, it would not simply be a case of a patient receiving a lethal prescription to use as and when they decide (as in Oregon and Victoria). The prescription would be delivered by a doctor (or authorised nurse) immediately before self-administration who would remain while the patient self-administered the medicine and has died (or decided not to take the medicine). The doctor/nurse would prepare the lethal dose for self-administration and, if necessary ‘prepare a medical device’ (a syringe and intravenous cannula) to facilitate this and ‘assist the person to ingest or otherwise self-administer the medicine’. As in Victoria, the process would consume many hours of medical time.

Judging by its website, *Dignity in Dying* seems to be favouring an ‘Oregon mark 2’ law. Like the Meacher Bill, this would include ratification by the High Court of all requests for PAS. However, it is not clear whether the lethal drug(s) would be delivered to the patient’s home for use ‘just in case’ as in Oregon or mimic the more complicated Meacher Bill.

Its website also states that ‘doctors, patients and the public need to have confidence that the law on AD will work in practice, will be safe and will remain unchanged’. In relation to this latter point, given all the available evidence, this is wishful thinking. The case for AD is stronger in relation to degenerative neurological disorders with a longer prognosis. Some people with progressive brain failure (dementia) will want to register for AD while they still have capacity, to be actioned when they are no longer able to interact meaningfully with those around them. However, this can lead to problems if they become distressed at attempts to administer oral or intravenous drugs [35].

## Conclusions

Any form of AD will have collateral harmful consequences for both medical care and society in general. The challenge is to find the ‘least worse’ option. A lack of readily available high-quality palliative care will always be coercive, there will always be abuse, the boundaries of the law will always be stretched, and a wrong diagnosis will mean that some people will die unnecessarily [12, 24].

It is imperative that conscientious objection by doctors and other healthcare professionals is guaranteed in law and fully respected in practice. PAS should *not* be seen as part of healthcare provision. The BMA also holds this view [28]. One way to achieve this would be for PAS to be delegated to a stand-alone *Department for Assisted Dying*, completely separate from the NHS and with its own budget. Victoria almost achieves this with its combination of Care Navigators, mandatory training for participating doctors, and a separate *Voluntary Assisted Dying Statewide Pharmacy Service*. It could be overseen by lawyers or judges and operated by trained technicians [36]. Doctors would be required only to confirm that a patient is medically eligible. Requests would be carefully processed without interfering in a patient’s clinical care, and in a way which would not undermine suicide prevention policies. Other options have proposed incorporating, for example, a review panel comprising a lawyer, a healthcare professional and an ethicist, backed up if necessary by an ombudsman [37, 38].

Further, alongside a de-medicalised model of AS, there is an urgent need to improve the provision of care for all terminally ill patients [39]. *Dignity in Dying’s* website states that ‘As well as campaigning to change the law on assisted dying we also support better end-of-life care which is accessible to all.’ Regrettably, there is no evidence of active campaigning on its part in this respect.

## Data Availability

All the data referred to are in the public domain.

## Declarations

### Author information

The author is one of the pioneers of hospice and palliative care in the UK, working first as Research Fellow in Therapeutics at St Christopher’s Hospice, London, in the early 1970s, followed by 25 years in Oxford at a hospice/palliative care unit in one of the University Hospitals. For many years he was Head of the WHO Collaborating Centre for Palliative Care. He has taught in over 40 countries and is the author or editor of several widely acclaimed textbooks, notably the Palliative Care Formulary.

## Abbreviations

AD: assisted dying
AS: assisted suicide
BMA: British Medical Association
DWDA: Death with Dignity Act (Oregon, USA)
E&W: England and Wales
GP: general practitioner (primary care doctor)
MAiD: medical assistance in dying
MND/ALS: motor neurone disease/amyotrophic lateral sclerosis
NHS: National Health Service (England and Wales)
NIV: non-invasive ventilation
PAS: physician-assisted suicide
PHC: palliative-hospice care
SAMS: Swiss Academy of Medical Sciences
UK: United Kingdom
VAD: voluntary assisted dying
